# *Listeria monocytogenes* Pathogenesis: The Role of Stress Adaptation

**DOI:** 10.3390/microorganisms10081522

**Published:** 2022-07-27

**Authors:** Thulani Sibanda, Elna M. Buys

**Affiliations:** 1 Department of Consumer and Food Sciences, University of Pretoria, Private Bag X20, Hatfield, Pretoria 0028, South Africa; thsibanda@gmail.com; 2 Department of Applied Biology and Biochemistry, National University of Science and Technology, Bulawayo P.O. Box AC939, Zimbabwe

**Keywords:** *Listeria monocytogenes*, virulence, stress response, Sigma factor B (SigB), positive regulatory factor A (PrfA)

## Abstract

Adaptive stress tolerance responses are the driving force behind the survival ability of *Listeria monocytogenes* in different environmental niches, within foods, and ultimately, the ability to cause human infections. Although the bacterial stress adaptive responses are primarily a necessity for survival in foods and the environment, some aspects of the stress responses are linked to bacterial pathogenesis. Food stress-induced adaptive tolerance responses to acid and osmotic stresses can protect the pathogen against similar stresses in the gastrointestinal tract (GIT) and, thus, directly aid its virulence potential. Moreover, once in the GIT, the reprogramming of gene expression from the stress survival-related genes to virulence-related genes allows *L. monocytogenes* to switch from an avirulent to a virulent state. This transition is controlled by two overlapping and interlinked transcriptional networks for general stress response (regulated by Sigma factor B, (SigB)) and virulence (regulated by the positive regulatory factor A (PrfA)). This review explores the current knowledge on the molecular basis of the connection between stress tolerance responses and the pathogenesis of *L. monocytogenes*. The review gives a detailed background on the currently known mechanisms of pathogenesis and stress adaptation. Furthermore, the paper looks at the current literature and theories on the overlaps and connections between the regulatory networks for SigB and PrfA.

## 1. Introduction

*Listeria monocytogenes* is a foodborne pathogen that is the causative agent of the human disease, listeriosis. It is primarily a ubiquitous environmental saprophyte found in many environmental niches such as water, soil, and vegetation [1]. Contaminated, often ready-to-eat (RTE) foods are the main transmission vehicles for human *L. monocytogenes* infections [2,3,4]. In most individuals, *L. monocytogenes* infections frequently result in mild, self-limiting febrile gastroenteritis [5]. However, in susceptible individuals, such as infants, the elderly, pregnant women and people who are immunocompromised, infections result in invasive listeriosis which has a high fatality rate of 20–30% [5]. As an invasive intracellular pathogen, *L. monocytogenes* depends on an arsenal of adhesion and invasion factors that facilitate its gastrointestinal tract (GIT) colonization and transit through the intestinal barrier [6]. Additionally, other virulence factors such as the cytolysin (listeriolysin O), actin polymerization protein ActA and phospholipases are important for the pathogen’s intracellular survival and spread of infection [7,8,9].

As a foodborne pathogen, *L. monocytogenes* encounters several physical and chemical stresses that impede its growth and survival along the food value chain [10]. In response to stress exposures, foodborne pathogens develop mechanisms to adjust cellular processes to a state that allows them to maintain viability and growth under stressful conditions [11]. The development of stress adaptive responses is a process that results from the sensing of environmental changes and reprogramming of gene expression towards the synthesis of stress response proteins that aid bacterial survival under harsh conditions [12]. This stress adaptation is the driving force behind the ability of *L. monocytogenes* to colonize and survive in different niches within food processing environments and to survive food processing and preservation hurdles [13].

In addition to bacterial stress adaptation being a necessity for survival in foods and the environment, some aspects of the stress responses are linked to bacterial pathogenesis [14]. Being an orally transmitted pathogen, *L*. *monocytogenes* must overcome the hostile host-defence systems in the human GIT as a first step to establishing a successful infection [14]. Some of these in vivo stress conditions such as acidic pH, increased osmolarity, and oxidative stress are conditions also encountered by the organism in foods and the environment [15]. Hence, food stress-induced adaptive tolerance responses to acid, osmotic and oxidative stresses can protect the pathogen against similar stresses in the GIT, and thus, directly aid its pathogenicity potential [16]. For some time, it has been known that the general stress response regulator (Sigma factor B, (SigB)) that controls the expression of genes for *L. monocytogenes* environmental stress adaptation, also controls the expression of some virulence factors involved in GIT colonization and pathogen internalization by the intestinal epithelium [17,18]. Besides the overlapping function of SigB, evidence has shown that the relationship between stress responses and virulence are intricately connected through direct and indirect interactions between the stress response regulator and the virulence regulator (positive regulatory factor A, (PrfA)) [19,20,21]. This review explores the current knowledge on the molecular basis of the connection between stress tolerance responses and the pathogenesis of *L. monocytogenes*. The review gives a detailed background on the currently known mechanisms of *L. monocytogenes* pathogenesis and the mechanisms of stress adaptation. Furthermore, the paper looks at the current theories and models explaining the connections between the stress response and virulence regulatory networks.

## 2. Overview of *L. monocytogenes* Infection Cycle

**Figure 1:**
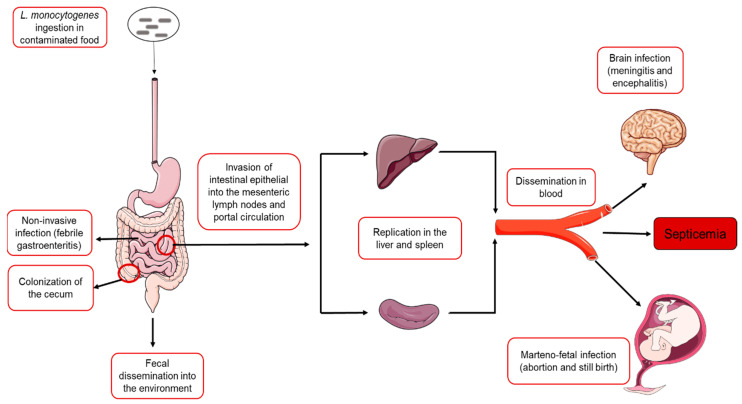
Schematic representation of the infection cycle of L. monocytogenes in humans. Following ingestion of contaminated food, the bacteria invade the intestinal barrier into the bloodstream. Through the portal circulation, the organism is transported to the liver and spleen where it multiplies before being disseminated into the bloodstream. The organism subsequently infects the brain and the placenta/fetus in pregnant women. The schematic art pieces were obtained from Servier Medical art (https://smart.servier.com (accessed on 1 March 2022)). Servier Medical Art by Servier is licensed under a Creative Commons Attribution 3.0 Unported License.

Through the ingestion of contaminated food, *L. monocytogenes* enters the GIT, where it can traverse the small intestines and establish a systemic infection that disseminates the pathogen to its main target organs (Figure 1). In most healthy individuals, *L. monocytogenes* infections remain largely extracellular within the intestinal lumen and often manifest with intestinal symptoms, typically as febrile gastroenteritis [6]. However, in susceptible individuals, the organism invades the epithelial barrier and crosses into the underlying lamina propria and mesenteric lymph nodes [22]. The organism is subsequently carried in the blood to the liver and spleen through the portal circulation [23]. In the liver and spleen, *L. monocytogenes* is first taken up by Kupffer cells and splenic dendritic cells as resident phagocytes in the respective organs [24,25]. Initially, the bacterial cells are contained within a membrane-bound vacuole inside the phagocytic cells. The bacteria subsequently lyse the vacuole and replicate within the cytosol of infected phagocytes before spreading into neighboring parenchymal cells [23]. In about 2–3 days following the initial phase of invasion, the bacterial multiplication inside the liver reaches maximum growth [23]. The susceptibility of the liver and spleen as the initial target organs for *L. monocytogenes* colonization and replication is thought to be a result of the fenestrated hepatic and splenic capillaries that permit an easy diffusion of *L. monocytogenes* from the bloodstream [23]. As replication niches, the liver and spleen act as reservoirs for *L. monocytogenes*, thus, enabling a re-seeding of the pathogen into the bloodstream, leading to the infection of additional organs [23]. In addition to establishing a systemic infection, studies in experimentally infected mice have shown that the organism can establish long-term colonization of the cecum and lumen of the colon, thus, creating a reservoir for the faecal spread of the organism back to the environment [26].

## 3. Pathogenesis of Invasive *L. monocytogenes* Infections

### 3.1. L. monocytogenes Virulence Factors

*L. monocytogenes* expresses several surface and soluble proteins that mediate the adhesion to target cells, internalization, intracellular multiplication and dissemination to other host cells [6]. The virulence factors are encoded either as separate loci across the bacterial genome or as clusters on pathogenicity islands [27]. A core of virulence genes (*prfA*, *hly*, *actA*, *plcA*, *mpl*, and *plcB*) encoded on the *Listeria* pathogenicity island 1 (LIPI-1) is conserved in the genomes of all *L. monocytogenes* strains [27]. Additionally, many other virulence factors encoded in separate loci, such as the internalin A/Internalin B (*inlAB*) operon, are also part of the virulence arsenal conserved in all *L. monocytogenes* strains [28]. The characteristics and roles of some of these proteins in the pathogenesis of *L. monocytogenes* are discussed in this section.

*Listeria adhesion protein (LAP)*. LAP is a 104-kDa cell wall protein ubiquitously found in all *Listeria* species [29]. It was first described by Pandiripally et al. [30] as protein p104 which was subsequently found to be an alcohol acetaldehyde dehydrogenase [31]. As an essential enzyme, LAP is produced primarily as a cytosolic protein in all *Listeria* species. However, in pathogenic species, the protein is translocated to the cell surface through the SecA2 secretory system to facilitate the adhesion of pathogenic *Listeria* species to intestinal cells [32,33]. The epithelial receptor for LAP is a constitutively expressed mitochondrial protein, heat shock protein 60 (Hsp60) [29]. In addition to acting as an adhesin, LAP has also been implicated in the translocation of the pathogen across the intestinal epithelium [34].

*Fibronectin binding protein (FbpA)*. Fibronectin binding proteins (Fbp) are cell wall-anchored proteins that are widely distributed in Gram-positive bacteria [35]. Fbps recognize and bind to fibronectin (a component of the human extracellular matrix that plays a role in inter-cellular interaction and adhesion) [36]. The interaction between bacterial Fbps and fibronectin molecules forms a three-component bridge (involving integrins), which facilitates the adhesion between the bacterial and the host cells [35]. The Fbp of *L. monocytogenes* (FbpA) was characterized by Dramsi et al. [37]. It is a 570-amino-acid polypeptide that shares a high homology to streptococcal Fbps (PavA of *Streptococcus. pneumoniae*, Fbp54 of *S. pyogenes* and FbpA of *S. gordonii*) [37]. However, unlike streptococcal Fbps, the *L. monocytogenes* FbpA is exposed on the surface of the bacterial cell without the signal peptide [37].

*Internalin A (InlA)*. InlA is one of the principal virulence factors of *L. monocytogenes* that was first described by Gaillard et al. [38]. It is an 80 kDa protein that is anchored onto the cell wall peptidoglycan through a C-terminal LPXTG motif [39]. InlA mediates the adhesion and internalization of the pathogen into enterocytes in the first step of invasion of the intestinal barrier [22]. An N-terminal leucine-rich repeat (LRR) domain acts as the recognition and binding site to the EC1 domain of the extracellular portion of E-cadherin [40,41].

*Internalin B (InlB).* InlB is another adhesion protein that plays a major role in *L. monocytogenes* binding to enterocytes and the subsequent invasion of the intestinal barrier [39]. Unlike InlA, InlB is anchored onto the cell wall through glycine and tryptophan (GW) modules that non-covalently interact with cell wall teichoic acids [42]. The LRR domain acts as the recognition and binding site to Met (a host receptor tyrosine kinase) [22]. *L. monocytogenes* also produces many other LRR proteins classified under the internalin family [43]. However, InlA and InlB have been identified as the principal adhesion proteins that mediate pathogen binding and invasion [44].

*Listeriolysin O (LLO)*. LLO is a 56 kDa pore-forming cytotoxin encoded by the *hly* gene [45,46]. It belongs to the family of cholesterol-dependent cytolysins (CDCs) [46]. It was one of the first *L. monocytogenes* virulence factors identified, based on the ability of virulent strains to cause hemolysis on blood agar [47]. Subsequent experiments identified the hemolysin as a sulfhydryl-activated toxin responsible for the intracellular growth of *L. monocytogenes* in human enterocyte-like Caco-2 cells [48,49]. The role of LLO is the lysis of the internalization vacuole, resulting in the release of the pathogen into the cytosol of host cells [50].

*Phospholipases.* Two types of phospholipases are required for *L. monocytogenes*. Phosphatidylinositol-specific phospholipase C (PI-PLC) is encoded by the *plcA* gene while phosphatidylcholine phospholipase C (PC-PLC) is encoded by the *plcB* gene [51,52]. PI-PLC plays a complementary role together with LLO in the lysis of the primary and secondary vacuole following pathogen internalization [44]. It catalyzes the cleavage of the membrane phosphatidylinositol into inositol phosphate and diacylglycerol [53]. PC-PLC is a broad-range phospholipase which is particularly required for the lysis of the double-membrane secondary vacuole and the primary vacuole in conditions of LLO deficiency [54]. PC-PLC is synthesized as a 33-kDa precursor that requires cleavage to produce the active 29-kDa enzyme [55]. A zinc-dependent metalloprotease (Mpl) encoded by the *mpl* gene is required for the maturation of PC-PLC [55].

*Actin-polymerizing protein ActA*. ActA is a surface protein encoded by the *actA* gene [56]. It mediates bacterial motility inside infected host cells through actin polymerization [56]. The protein is anchored on the bacterial cell membrane through its hydrophobic C-terminal domain while the functional N-terminal domain is exposed to the host cell cytoplasm [56]. Within the bacterial cell surface, ActA exhibits an asymmetrical distribution, being more concentrated at one polar end of the cell. The asymmetrical distribution is responsible for the directionality of *L. monocytogenes* motility [57,58]. To facilitate intracellular motility, ActA mediates actin nucleation and filament formation through the recruitment of host vasodilator-stimulated phosphoprotein (VASP) and actin-related proteins-2 and 3 (Arp2/3) complex [59,60].

### 3.2. Gastrointestinal Tract Colonization and Invasion of Host Cells

Due to its severity and high fatality rates, much of the focus on the pathogenesis of listeriosis is placed on invasive infections. However, evidence shows that non-invasive listerial febrile gastroenteritis outbreaks are very common [61,62,63,64]. Non-invasive *L. monocytogenes* infections are typically characterized by enteric symptoms such as vomiting, non-bloody diarrhea, nausea and fever that occur within a short period (24 h) following the ingestion of contaminated foods [62,65]. The mechanisms underlying the pathogenesis of non-invasive *L. monocytogenes* infections remain unclear [65]. Recently, a few studies have attempted to elucidate the mechanisms of *L. monocytogenes* gastrointestinal tract colonization [26,65]. Based on in vitro and mice models, the actin polymerization protein ActA—which mediates the cell-to-cell spread of the pathogen in invasive listeriosis—has also been implicated in intestinal colonization [26]. Using *actA* gene mutants in orally infected mice, Travier et al. [26] found that ActA can mediate *L. monocytogenes* aggregation both in vitro and in the gut lumen. The postulated mechanism of the ActA-mediated aggregation is based on direct ActA–ActA interactions through the C-terminal regions (which are not involved in polymerization) [26]. In the same study, the researchers found that ActA-dependent aggregation was also responsible for an increased ability to persist within the cecum and colon lumen of mice. Additionally, Halbedel et al. [65] observed a genetic correlation between the *L. monocytogenes* disease outcome (invasive or non-invasive) and the presence or absence of a functional chitinase gene (*chiB*) in which gastroenteritis outbreak isolates possessed a premature stop codon in the *chiB* gene. However, the restoration of chitinase production in a non-invasive isolate could not generate the invasiveness characteristic [65].

**Figure 2:**
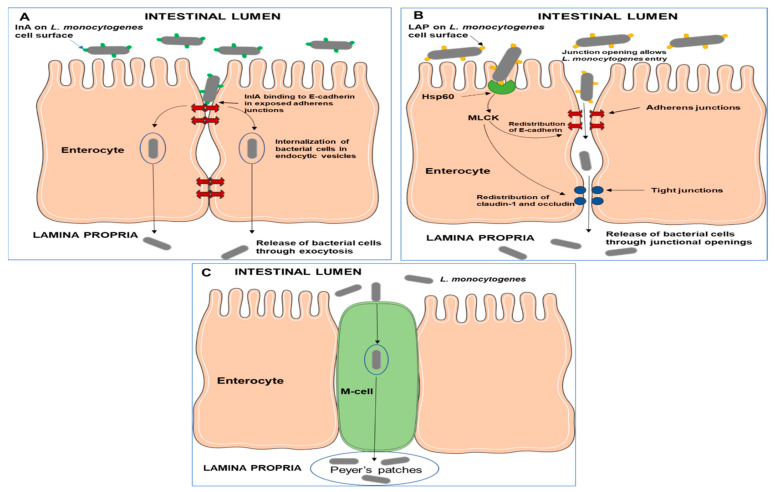
Pathways of the *L. monocytogenes* invasion of the intestinal barrier. In the InlA-mediated transcytosis (**A**), bacterial cells are taken into the enterocytes by endocytosis through the interaction of InlA and any exposed E-cadherin receptors followed by subsequent exocytosis into the lamina propria. In the LAP-mediated translocation (**B**), the interaction between the bacterial surface protein LAP and the receptor Hsp60 causes an opening of tight junctions facilitating the movement of bacterial cells into the lamina propria. In the M-cell-mediated pathway (**C**), bacterial cells are passively taken in by M-cells and released by exocytosis into the lamina propria. The schematic art pieces were obtained from Servier Medical art (https://smart.servier.com). Servier Medical Art by Servier is licensed under a Creative Commons Attribution 3.0 Unported License.

The first step in the pathogenesis of invasive listeriosis is the ability of the pathogen to cross the intestinal epithelial barrier. Although the complete mechanisms are still not fully understood, three well-elucidated pathways have thus far been used to explain the process [22]. These three pathways (Figure 2) are the InlA-mediated transcytosis, the LAP-mediated translocation, and the microfold (M-cell)-mediated transcytosis [22].

*InlA-mediated transcytosis.* The InlA- mediated pathway (Figure 2) is the primary route by which *L. monocytogenes* invades intestinal cells. InlA is a cell wall-anchored protein that mediates the uptake of *L. monocytogenes* into non-phagocytic cells through receptor-mediated endocytosis [66]. InlA promotes pathogen adhesion and the invasion of the intestinal epithelium through an interaction with its receptor, E-cadherin (a component of adherens junctions) [44]. Adherens junctions, tight junctions, and desmosomes are part of the apical junctional complex that provides a paracellular seal between adjacent epithelial cells [22]. The InlA interaction with receptors occurs at sites where E-cadherin is transiently exposed to the intestinal lumen [67,68]. The transient exposure of E-cadherin occurs during cell extrusion and junction remodeling [68]. Furthermore, changes in the shape of goblet cells can also result in the exposure of the E-cadherin component of the cell junctions [67]. Through interaction with the receptor, bacterial cells are taken into the enterocytes by endocytosis and are subsequently then released into the lamina propria by exocytosis [22]. The binding of InlA induces the recruitment of other junctional proteins, α-catenin and β-catenin, as well as actin and p120 catenin, which facilitate E-cadherin clustering at the site of bacterial entry [69]. Subsequently, a post-translational modification of E-cadherin (phosphorylation by the tyrosine kinase, Src and ubiquitination by the ubiquitin-ligase Hakai) induces endocytosis through caveolin or clathrin [22,69]. Ultimately, the InlA/E-cadherin-mediated endocytosis involves components of the host cytoskeleton that facilitate the formation of localized host cell membrane protrusions that force the formation of endocytic vesicles around the adherent bacteria cell [44]. It is now known that host cytoskeletal proteins involved in actin nucleation such as the Arp2/3 complex and VASP are activated in response to InlA binding to its receptors [39,70].

Unlike InlA, InlB does not play a major role in the invasion of intestinal cells [39]. However, together with InlA, it plays a role in the invasion of other tissues such as the liver, spleen, CNS and placenta [23]. The InlB receptor is the ubiquitous tyrosine kinase Met whose normal ligand is Hepatocyte Growth Factor (HGF) [44]. The binding of InlB to Met results in the autophosphorylation of the cytoplasmic tail of the Met proteins, initiating a reaction cascade that culminates in the localized polymerization of actin and internalization of bacterial cells in the same way as InlA [66].

*LAP-mediated translocation*. For a long time, the InlA-mediated pathway was established as the main route of *L. monocytogenes* traversal of the intestinal epithelium [67,68,69]. However, subsequent evidence that strains possessing non-functional InlA could cause infections in orally dosed mice and guinea pigs [71,72] showed that the pathogen can use alternative mechanisms to achieve intestinal invasion [34]. The surface protein, LAP, which was initially identified as an adhesin that facilitates the binding of *L. monocytogenes* to enterocytes, also contributes to the translocation of the pathogen across the intestinal epithelium [34]. The pathway of LAP-mediated invasion (Figure 2) was elucidated by Drolia et al. [34] using a Caco-2 cell line and a mouse model. The researchers showed that LAP induces the intestinal epithelial barrier dysfunction as a mechanism of promoting bacterial translocation. The binding of LAP to its luminal receptor protein Hsp60 activates myosin light-chain kinase (MLCK) that mediates the opening of the intestinal barrier through the redistribution of junctional proteins, claudin-1, occludin, and E-cadherin [34]. These reactions cause the opening of tight junctions between neighboring enterocytes allowing *L. monocytogenes* translocation [22,34]. Furthermore, the LAP-mediated translocation is thought to be an important precursor event for the InlA-dependent invasion, as it potentially provides pathogen access to E-cadherin in exposed adherens junctions [34].

*M-cell mediated transcytosis*. The microfold (M) cells are specialized epithelial cells that survey the intestinal mucosa for any antigens as part of the mucosal immune response. They readily take up antigens from the intestinal mucosa and transcytose them across the intestinal epithelium to the lymphoid tissues of the Peyer’s patches [73]. This process also serves as a passive route for the transcytosis of pathogens into the basolateral side of the follicle-associated epithelium [74]. While the role of M-cells in the transcytosis of *L. monocytogenes* has been well established, the mechanism of the pathogen interaction with such cells is not fully understood [74]. Evidence from in vitro and orally infected mice models has shown that in the absence of InlA, *L. monocytogenes* rapidly accumulate in the Peyer’s patches [75,76]. The prevailing paradigm on the M-cell mediated pathway is that transcytosis occurs across the M cells through a vacuole [22,23]. However, Rey et al. [74] established that in addition to the rapid vacuolar transcytosis, *L. monocytogenes* also escapes to the cytosol of the M-cells by vacuolar rupture. Once in the M-cell cytosol, the pathogen can initiate a direct ActA-based M-cell-to-enterocyte spread [74].

### 3.3. Intracellular Survival and Dissemination

The ability to cross the intestinal barrier provides the main gate of *L. monocytogenes* entry into the bloodstream. Due to its predilection for the CNS and the placenta in pregnant women, neurolisteriosis, maternofetal infection and septicemia are the main clinical manifestations of invasive listeriosis [77]. The high tropism of *L. monocytogenes* for these tissues is unclear. The possible explanation has been attributed to the presence of E-cadherin and Met, the two receptor proteins for InlA and InlB, respectively [7]. Because of the presence of Met in the human umbilical vein endothelial cells (HUVEC), *L. monocytogenes* can invade the human placenta through an InlB-dependent mechanism [78]. In the CNS, both receptors are expressed at the surface of choroid plexus epithelial cells and Met is additionally expressed at the brain endothelial cells of the blood-cerebrospinal fluid (CSF) and blood–brain barriers. Hence, the invasion of the CNS is facilitated by both InlA and InlB mechanisms [7].

**Figure 3:**
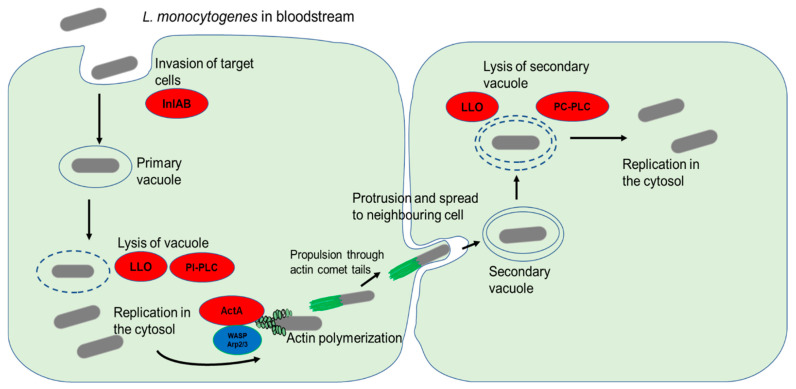
*L. monocytogenes* invasion of target cells and cell-to-cell spread. The bacterial surface internalins InlA and InlB interaction with their respective cell surface receptors result in the internalization of bacterial cells. The primary endocytic vacuole is then lysed through the activity of LLO and PI-PLC. Following a period of replication in the cytosol, the release of ActA stimulates actin polymerization by recruiting host nucleation proteins VASP and Arp2/3 complex. The formation of comet tails propels the bacterial cells and enables them to spread to neighboring cells through membrane protrusions. Lysis of the double membrane of the secondary vacuole by the action of LLO and PC-PLC causes the release of bacterial cells into the cytosol.

Once internalized into the target cells in a primary vacuole, the next step in the infection cycle is the escape from the primary vacuole into the cell cytosol [79] (Figure 3). This vacuolar escape is mediated by the production of LLO [8,79]. This pore-forming cholesterol-dependent cytotoxin causes the rupture of the vacuole and release of the bacterial cells into the host cell cytosol [80]. In addition to LLO, *L. monocytogenes* also employs phospholipases, such as PI-PLC, that significantly enhance the lysis of the primary vacuole [52]. Following a period of intracellular replication inside infected cells, the production of ActA results in the formation of actin comet tails which facilitate bacterial motility inside the cells as well as the spread to uninfected cells through membrane protrusions [9]. The double membrane of the resulting secondary vacuole is degraded by LLO in collaboration with PC-PLC [9].

### 3.4. Clinical Outcomes of Invasive L. monocytogenes Infections

The clinical outcomes of listeriosis depend on the health status of the infected individual and are often correlated to underlying factors and comorbidities such as cancer, chronic renal, cardiovascular, and liver disease, multi-organ failure, and old age [81,82,83]. In neurolisterial infections, the most common symptoms include meningitis, meningoencephalitis, and rhombencephalitis [7]. For maternofetal listeriosis, the main clinical features include amniotic inflammation (amnionitis), preterm labor, stillbirths, and spontaneous abortions. In severe cases, widespread micro-abscesses and granulomatosis infantiseptica in newborns can occur [84]. Fever, diarrhea, influenza-like symptoms, multi-organ failure, and decompensated comorbidities are the most commonly reported clinical features associated with listerial septicemia [81]. In rare cases, infections can also affect a variety of organs and organ systems [85]. These infections normally involve the cardiovascular system (endocarditis) [86], respiratory tract infections (pleural infections and pneumonia) [87], biliary tract infections (cholecystitis, cholangitis, and biliary cyst infection) [88], and bone and joint infections, especially those involving orthopedic implant devices [89].

## 4. *L. monocytogenes* Stress Responses and Adaptation

Similar to all living organisms, the ability of *L. monocytogenes* to sense and respond to environmental changes is essential to its survival. Environmental conditions such as osmotic pressure shifts, temperature shifts, pH extremes, changing redox potential and fluctuating nutrient availability impose stress on microbial cells [10]. At their extremes, stress conditions can cause damage to the cellular structural components and disrupt the homeostatic balance inside microbial cells, resulting in cell death [11]. In many instances, microorganisms are exposed to mild stress levels that only reduce growth without causing loss of viability [90]. Due to the demand for minimally processed foods that preserve the natural freshness and nutritional quality, many foods are processed by the use of mild processing technologies that apply a combination of many sub-lethal stress treatments [91]. Despite the benefits of such approaches, exposure to sub-lethal stresses can induce the development of adaptive stress tolerance responses that enhance the survival of pathogens when exposed to subsequent lethal stress along the food value chain [15]. Many sub-lethal stress hurdles employed in food preservation have been proven to induce adaptive tolerance to lethal stress treatments in *L. monocytogenes* [92,93,94,95,96]. Exposure to sub-lethal acid at pH 5.0, sub-lethal heat at 46 °C, and sub-lethal H_2_O_2_ at 100 ppm H_2_O_2_ induces tolerance to lethal acid at pH 3.5; lethal heat at 63 °C and lethal H_2_O_2_ at 1000 ppm, respectively [95,97,98]. In addition to homologous adaptive responses, heterologous cross-adaptation between different stress factors also occurs [99]. For example, protection against lethal acid stress in *L. monocytogenes* can be induced by sub-lethal NaCl and heat stress exposures [96,97,98]. The development of adaptive responses has implications for pathogen survival in foods and, subsequently, in the GIT.

### 4.1. Adaptation to Stress in Foods and Food Processing Environments

The mechanisms responsible for the development of *L. monocytogenes* adaptive tolerance responses against the common environmental and food-related stresses (acid, osmotic, heat, cold, oxidative stress) have been elucidated [95,100,101,102,103,104]. Upon exposure to stress, *L. monocytogenes* modulates the transcription of stress response genes whose effect is to trigger cellular processes that allow the pathogen to survive and grow in the presence of stress [21]. The specific mechanisms of response to acid, osmotic, heat, cold, and oxidative stresses are briefly described in this section.

#### 4.1.1. Osmotic Stress Adaptation

Osmotic stress results from changes in environmental osmolarity that disrupt the cellular osmotic balance [105]. Changes in environmental osmolarity can result from environmental salinity, desiccation and the use of hyperosmotic solutes in foods [106,107]. Extreme hyperosmotic conditions result in loss of cell turgor and cell death due to plasmolysis [105]. The ability of *L. monocytogenes* to withstand hyperosmotic conditions has been known for a long time [98,108]. In response to hyperosmotic conditions, the organism actively accumulates compatible solutes as a way of counterbalancing the negative effects of outward water movement. Compatible solutes are low-molecular-weight, highly soluble compounds that bear a neutral charge at physiological pH, whose accumulation inside the cells helps in restoring cell turgor, without affecting cytoplasmic function [109]. Although several compounds have been identified as potential osmoprotectants, glycine betaine (*N*,*N*,*N*-trimethylglycine) and carnitine (β-hydroxy-γ-*N*-trimethyl aminobutyrate) are the most potent at conferring osmoprotection on *L. monocytogenes* [110]. Notably, both compounds are not synthesized by *L. monocytogenes* but are fairly ubiquitous in foods of both plant and animal origin and, therefore, their intracellular accumulation is achieved by active transport from the environment [109,111].

The osmotic stress response is triggered by changes in osmotic pressure as the main signal [112]. However, the mechanism of signal sensing and transduction has only recently been elucidated [113]. The modulation of osmolyte transport and the expression of genes encoding osmolyte transporters is regulated Cyclic di-AMP (c-di-AMP) [105,113]. Two glycine betaine transporters (Gbu and BetL) and a single carnitine transporter OpuC have been known to respond to osmotic upshifts [109]. Gbu is an ATP-dependent transporter encoded by the *gbu* operon that mediates the uptake of glycine betaine in response to osmotic and cold stress [114]. BetL is a non-ATP-dependent secondary transporter encoded by *betL*, whose uptake of glycine betaine is coupled to Na^+^ symport [115]. Carnitine transport is mediated by the ATP-dependent transporter, OpuC, a product of the *opuC* operon that responds to both osmotic and cold stress [116].

#### 4.1.2. Acid Stress Adaptation

Organic acids constitute one of the most frequently used preservatives in foods. Although there are several antimicrobial targets of acids [117,118,119], the primary antimicrobial effects result from the protonation of the cytoplasm and disruption of intracellular pH [120]. Two amino acid decarboxylation systems (the glutamate decarboxylase (GAD) and arginine deiminase (ADI)) have been known to protect *L. monocytogenes* against acid stress [102,104].

The GAD system depends on the enzyme glutamate decarboxylase, a product of the *gadD* operon which decarboxylates glutamate to produce γ-amino butyric acid (GABA) while consuming a proton and releasing a bicarbonate anion. The decarboxylation is coupled with an antiporter (GadT) that takes out the produced GABA while taking in glutamate [104]. *L. monocytogenes* produces three glutamate decarboxylase enzymes (GadD1, GadD2, and GadD3) and two antiporters (GadT1 and GadT2) that are encoded as pairs consisting of *gadD1T1* and *gadD2T2* operons in separate parts of the genome [104]. The genes have distinct functions in the acid stress response of *L. monocytogenes*. The expression of *gadD1T1* is required for mild acid (pH 5.1) survival, while the *gadD2T2* expression is needed for severe acid stress (pH 2.8), and therefore, is necessary for the adaptive acid tolerance response (ATR) [121].

The ADI system involves the conversion of arginine to ornithine accompanied by the production of ammonia and carbon dioxide [122]. As part of the system, an arginine-ornithine antiporter (protein ArcD encoded by *arcD*) facilitates the uptake of arginine in exchange for ornithine. Once inside the cell, the deimination of arginine by the enzyme arginine deiminase (encoded by the *arcA*) produces ammonia and citrulline. The latter is then converted to ornithine and carbamoyl phosphate through the enzyme ornithine carbamoyltransferase (encoded by *arcB*). Carbamoyl phosphate is subsequently converted to ammonia and carbon dioxide through the activity of carbamate kinase (encoded by *arcC*) [102]. This reaction reduces internal pH through the conversion of ammonia (NH_3_) to ammonium ions (NH_4_^+^) [123].

#### 4.1.3. Heat Stress Adaptation

Thermal processing is an established method of food preservation, known for its lethality, especially at elevated temperatures. As a non-spore-former, *L. monocytogenes* is generally susceptible to heat stress, although it has been reported to exhibit thermotolerance upon exposure to heat shock at sublethal temperatures [124,125]. Moreover, heat resistance can be induced by exposure to acid, oxidative, alkali, and chlorine stresses [126,127]. The heat stress response is universal in all prokaryotes and is triggered by temperature up-shifts above the normal growth range. Its main effect is the protection of cellular proteins and enzymes against heat-induced denaturation that affects their physiological functions [101]. The heat shock response of *L. monocytogenes* involves the increased transcription of heat shock genes coding for three classes of heat shock proteins (HSPs) [128]. Of these three classes of HSPs, the expression of Class I and Class III proteins is a direct response to heat stress, while Class II proteins are general stress response proteins under the regulatory control of σ^B^ [101]. Class I HSPs are chaperones made up of the proteins, dnaK, dnaJ, groES, and groEL encoded in two operons (the *dnaK* and *groEL-groES* operons) [129,130]. Class III HSPs are ATP-dependent proteases (clpP, clpC, clpE, and clpB) involved in the proteolysis of misfolded proteins [13]. Under normal growth temperature, the expression of the Class I HSPs and Class III HSPs genes is prevented by HrcA and CtsR repression, respectively [101,131]. Under conditions of elevated temperatures, de-repression is achieved through the reduced DNA binding of repressors and improved binding of sigma factor A, leading to increased transcription [101].

#### 4.1.4. Cold Stress Adaptation

Although *L. monocytogenes* is typically a mesophile, with an optimum growth temperature of 30–37 °C, the organism has a remarkable ability to multiply under low-temperature conditions [132]. While part of this psychrotrophic growth ability may be intrinsic, a significant part of it is induced by exposure to cold conditions [133]. At least three mechanisms are utilized by *L. monocytogenes* in response to cold stress-imposed challenges. These include the adjustment of the fatty acid composition of the cell membranes in order to maintain fluidity, the increased expression of cold shock proteins (CSP), and the accumulation of osmolytes and oligopeptides [103].

Upon exposure to low temperatures, the membrane of mesophilic bacteria changes from an elastic liquid crystalline state to a gel-phase state, resulting in the impairment of nutrient uptake [100]. The low-temperature growth propensity of *L. monocytogenes* is related to its ability to maintain membrane fluidity, which is necessary for nutrient transport [134]. The mechanism of cold stress adaptation involves the adjustment of membrane fluidity through the incorporation of unsaturated anteiso-branched-chain fatty acids (BCFA) [135]. Anteiso-BCFAs have a lower melting point than the analogous iso-BCFAs that account for >95% of the membrane fatty acid composition of *L. monocytogenes* cells growing at 37 °C [136]. A key determinant in the adjustment of membrane fluidity is the enzyme β-ketoacyl-acyl carrier protein synthase III (FabH) which catalyzes the initial condensation reaction between iso- and anteiso-branched α-keto acids and acetyl-coenzyme A [135,137].

CPSs belong to a family of small highly conserved and structurally related proteins that are widely distributed in the prokaryotic kingdom [100]. Within the genomes of *L. monocytogenes*, three families of CSP genes (*CspA*, *CspB*, and *CspD* coding for CpsA, CpsB and CpsD, respectively) have been found [138]. While these proteins are dispensable for growth at 37 °C, they are required for growth at 5–10 °C [139,140]. Using directed mutagenesis, Schmid et al. [139] observed that CspA is the main CSP required for low-temperature growth of *L. monocytogenes*. Although the exact functions of CSPs are still to be fully elucidated, the current postulation is that these proteins act as nucleic acid chaperones that bind RNA and DNA, thus, facilitating the control of processes such as replication, transcription, and translation within bacterial cells under cold stress [100]. This is presumably necessary to help the organisms overcome the challenges of DNA and RNA supercoiling, which is associated with low-temperature growth [100], coupled with the role of CSPs are RNA helicases, that bind to ribosomes and facilitate RNA maturation challenges at low temperatures [141]. Four DEAD-box RNA helicase genes have been found in the genome of *L. monocytogenes* [142]. Using knock-out mutants, the helicases were found to be necessary for *L. monocytogenes* cold growth [141,143].

*L. monocytogenes* cold stress adaptation also involves the accumulation of compatible solutes, glycine betaine, and carnitine, as well as oligopeptides as cryoprotectants [103]. The main osmolyte transporters Gbu, BetL and OpuC induced by osmotic stress are also induced by cold stress [144,145]. The accumulation of oligopeptides in *L. monocytogenes* is mediated by the oligopeptide permease transporter (OppA) encoded by the *opp* operon [146]. The exact roles of the accumulated osmolytes and oligopeptides in the cold stress response are unclear. Some posited roles include acting as cryoprotectants and stabilization of enzymes [103].

#### 4.1.5. Oxidative Stress Adaptation

Oxidative stress results from the production and accumulation of reactive oxygen species (ROS) such as superoxide anion, hydrogen peroxide, hydroxyl radicals, peroxyl radicals, and singlet oxygen that cause damage to cellular molecules such as DNA, lipids, and proteins [147]. As a facultative anaerobe, *L. monocytogenes* is oxidative stress-tolerant. Catalase and superoxide dismutase encoded by the *kat* and *sod* genes, respectively, are the primary antioxidant enzymes produced by *L. monocytogenes* [148,149]. The two enzymes detoxify the superoxide anion and hydrogen peroxide generated by aerobic metabolism [150]. In addition to the detoxifying enzymes, *L. monocytogenes* possesses a metal-dependent peroxide sensor, PerR (peroxide repressor), that regulates the expression of peroxidase genes and genes for metal homeostasis [151]. The PerR regulon includes *kat*, *fur* (iron homeostasis regulator), *hemA* (haem biosynthesis), *fri* (iron-binding protein) and *fvrA* (iron efflux pump) [151,152].

### 4.2. Adaptation to Stress in the GIT

The successful colonization and subsequent GIT invasion by *L. monocytogenes* rely on the ability of the pathogen to overcome the harsh conditions associated with the innate defences of the GIT. The first physical stress encountered by *L. monocytogenes* in the GIT is the low pH of the stomach that the pathogen must deal with as it transits to the small intestines [153]. The critical role of acid stress adaptation in *L. monocytogenes* pathogenesis has been demonstrated through in vitro infection models of enterocyte-like cells and mice models [154,155]. The survival of stomach acidity is attributable to the expression of the GAD system [104]. The *gadD2T2* operon, which is responsible for the ATR of *L. monocytogenes* induced by environmental acid stress exposure, is required for the survival in gastric fluid [104,121].

Once *L. monocytogenes* passes through the low pH of the stomach, it is faced with high osmotic stress and bile stress in the lumen of the small intestines [156]. The resistance to osmotic stress in the GIT has been attributed to the activation of the carnitine transporter opuC [116,140]. *L. monocytogenes* mutants with *opuC* gene deletions have been shown to exhibit limited pathogenicity in animal infection models, while the deletion of glycine betaine transporter genes *gbu* and *betL* do not seem to affect virulence [116,140]. The importance of carnitine as the preferred osmolyte in the GIT survival of *L. monocytogenes* is probably linked to its relative abundance in mammalian tissues [140]. Bile stress tolerance is a critical factor in *L. monocytogenes* GIT survival and colonization. The pathogen produces a bile salt hydrolase (BSH) enzyme (encoded by *bsh*) that catalyzes the hydrolysis of the amide bond between the bile acids (cholic acid and chenodeoxycholic acid) and the amino acid conjugates [157]. The enzymatic hydrolysis is complemented by an increased expression of the transporter protein BilE (a product of the *bilE* gene) which is responsible for bile exclusion [158].

## 5. Crosslink between Stress Responses and Virulence

### 5.1. Regulation of L. monocytogenes Stress Response

In *L. monocytogenes*, the general stress response alternative sigma factor B (SigB) modulates a reprogramming of gene expression that facilitates the survival and protection against harsh environmental conditions [12]. Since it was first described in *L. monocytogenes* three decades ago [159], several roles of SigB have been identified [160,161,162,163]. The identified regulon of this general stress response regulator encompasses more than 200 genes that are involved in environmental stress survival, metabolism, and virulence [20,164]. As a general stress response regulator, SigB mediates survival under a broad range of lethal stresses along the food value chain. Transcriptomic and mutagenesis experiments have shown that responses to the common environmental and food stress factors (acid, osmotic, heat, cold, oxidative stress and nutrient stress) are sigB-dependent [20,159,165,166,167]. Although SigB is the central transcriptional regulator of stress survival genes in *L. monocytogenes*, some alternative transcriptional regulators are also utilized in response to specific stress factors. For instance, the expression of Class I and Class III *hsp* genes relies on HrcA and CtsR proteins as negative regulators [101], while the peroxide repressor PerR regulates the expression of oxidative stress response genes [151].

In addition to regulating stress responses in the environment, SigB also modulates gene expression in response to stress conditions encountered along the oral infection route. *sigB* deletion mutants exhibit limited pathogenicity in orally infected model animals [168]. The SigB modulation of the *gadD2T2* operon, *opuC*, *bsh* and *bilE* gene expression is critical to the survival of stomach acidity, intestinal osmotic and bile stresses, respectively, as a prerequisite for a successful intestinal invasion [116,121,157,158]. Besides its role in stress survival and adaptation, evidence shows that SigB also plays a critical role in the invasion of the intestinal barrier and initiation of infection [12,21]. Thus, the general stress response regulator facilitates a smooth transition from the environmental saprophytic life cycle to the pathogenic life cycle inside host cells.

#### Molecular Mechanisms of SigB-Dependent Regulation

**Figure 4:**
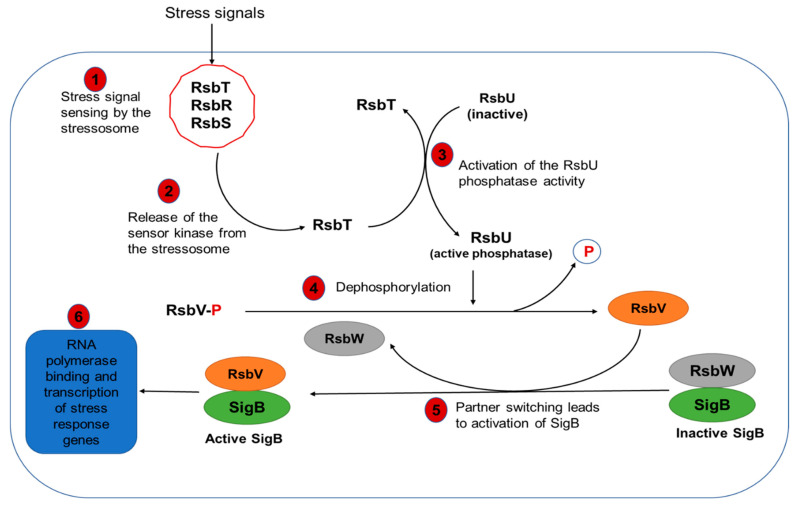
Mechanism of Rsb-mediated regulation of SigB expression and activity in *L. monocytogenes*.

The SigB protein is encoded by the *sigB* gene as part of an operon that includes seven other genes referred to as the regulation of sigma B (*rsb*) genes (*rsbR*, *rsbS*, *rsbT*, *rsbU*, *rsbV*, *rsbW*, and *rsbX*) coding for Rsb proteins [169]. The Rsb proteins are responsible for the detection of environmental stress signals and the regulatory cascade that controls the activity of SigB. The sensing and transduction of environmental signals is mediated by a 1.8 MDa supra-macromolecular stressosome consisting of a complex of RsbR-RsbS-RsbT proteins [12,170]. Although the exact mechanisms of stress sensing are not clearly understood, the current model is based on phosphorylation events by the sensor kinase RsbT and its subsequent release from the stressosome complex (Figure 4). RsbT subsequently activates RsbU, converting to an active phosphatase. Through its dephosphorylation activity, RsbU in turn activates RsbV [12,171]. In exponentially growing cells, the SigB protein exists as an inactive form bound to the anti-SigB protein, RsbW [169]. The activation of SigB is achieved by a partner-switching mechanism in which the release of RsbW is mediated by the binding of the dephosphorylated form of the anti-SigB protein, RsbV (Figure 4) [169].

### 5.2. Regulation of L. monocytogenes Virulence

The transition of *L. monocytogenes* from an environmental saprophyte to an intracellular pathogen is facilitated by changes in gene expression patterns from environmental survival-related genes to intracellular survival-related genes. Central to the transition is the role of the protein PrfA, encoded by the *prfA* gene [172]. PrfA is a 27 kDa member of the cyclic AMP receptor protein (Crp)/fumarate nitrate reductase regulator (Fnr) family of bacterial transcription factors [28,173]. The Crp/Fnr transcriptional activators are symmetrical homodimers consisting of an N-terminal cAMP binding domain and C-terminal DNA binding domain [28]. Its regulon includes a block of virulence genes (*hly*, *actA*, *plcA*, *mpl*, and *plcB*) encoded by the *Listeria* pathogenicity island 1 (LIPI-1) and the *inlAB* operon on a separate chromosomal locus [172,174,175]. Apart from the invasion proteins, inlA and inlB, the role of PrfA in *L. monocytogenes* pathogenesis is largely on the expression of virulence factors associated with the intracellular stage of the infection as well as the cell-to-cell spread [19,176]. Hence, the transcriptional regulator is activated once the pathogen is in the cytosol of host cells, switching the bacterial cells from the avirulent to the virulent state [176]. The signals and mechanisms that trigger *prfA* expression in the host cytosol have only become clearer in the last decade [177,178,179]. Branched chain amino acids (BCAA) and phosphorylated hexoses have been identified as the metabolic signals inside host cells [180,181]. An exception to the need for intracellular activation has been observed with *L. monocytogenes* mutants that constitutively express the *prfA* gene (prfA* mutants) [182,183]. Such prfA* mutants exhibit a high virulence in animal infection models. However, their environmental stress survival ability is poor [176]. PrfA activates transcription by binding to the PrfA box, a 14-bp A/T-rich palindromic nucleotide sequence located ~40 bp upstream of the transcriptional start sites of genes under its control [21].

### 5.3. Regulatory Intersection between Stress Response and Virulence

**Figure 5:**
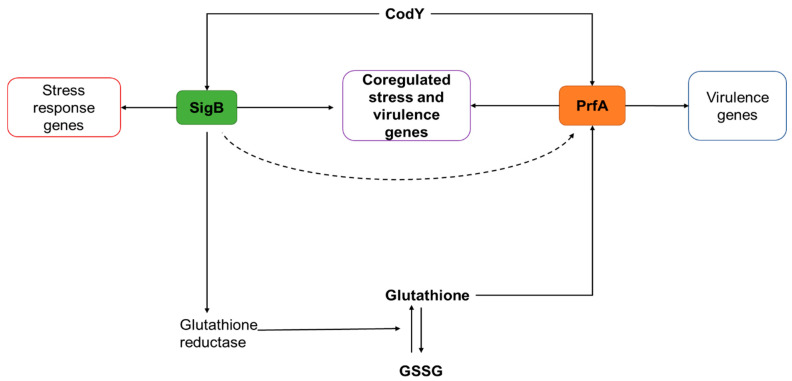
An illustration of the overlap and interactions between SigB and PrfA in *L. monocytogenes*. Solid arrows indicate positive regulation. The dotted arrow indicates negative regulation.

A plethora of evidence has shown that responses to stress in *L. monocytogenes* influence pathogenesis [150,154,184,185,186]. In most reports, the relationship between stress response and pathogenesis is attributed to adaptive tolerance responses that enable the pathogen to survive host innate defences in the GIT [15,16]. Along with the adaptive stress tolerance, the relationship can also be attributable to overlaps and direct interaction between the regulatory networks of stress and virulence [12,21]. Some of the currently understood mechanisms behind the interplay between the stress and virulence regulatory networks are described in this section. An illustration of the overlap and interactions between transcriptional regulators of stress and virulence is depicted in Figure 5.

*Coregulation of the inlA/B operon, actA and bsh:* The dispensability of SigB in the pathogenesis of *L. monocytogenes* is a fact that has been established for a long time [18,168]. The initial evidence for the role of SigB in *L. monocytogenes* pathogenesis was based on observations that *sigB* deletion mutants are avirulent on oral infection but are fully virulent when injected intravenously [168]. Subsequent elucidation of the role of SigB established that in addition to its stress regulon, this transcriptional regulator extends to the control of virulence genes *inlA*, *inlB*, and *actA* (Table 1) [58,187]. The overlapping regulatory controls of SigB and PrfA on the *inlAB* operon provide the connection between the stress response and virulence of the pathogen [156]. The biological significance of this coregulation of the *inlAB* operon has been explained in terms of the need to facilitate a transition from GIT survival to the invasion of the intestinal barrier, in which SigB is necessary for initiating infection in the intestinal phase before yielding the regulatory function to PrfA in the intracellular stages of infection [156]. Moreover, PrfA also extends its regulatory network to some stress response genes involved in the GIT survival phase (Table 1), such as the *bsh* gene [184,188]. Guariglia-Oropeza et al. [184] suggested a model where SigB-dependent gene expression plays a role in the survival of acid and osmotic stress exposures in the early stages of GIT infection before the subsequent induction of PrfA by the exposure to bile. This intestinal expression of PrfA regulon potentially primes *L. monocytogenes* for the subsequent intracellular stage of infection. While ActA is primarily a virulence factor necessary for the intracellular stage of *L. monocytogenes* infection, it is also produced in the extracellular environment, where it mediates aggregation and biofilm formation [26,58]. Within the intestinal lumen, *actA* expression is under the dual regulation of both SigB and PrfA and is necessary for GIT colonization [26,189].

*SigB downregulation of prfA expression and maintenance of basal levels:* A growing body of evidence indicates that the interactions between SigB and PrfA extend beyond the coregulation of the *inlAB* operon (Figure 5) [19,191]. Three transcriptional promoters (*prfA*P1, *prfA*P2 and *prfA*P3) are utilized in the control of *prfA* expression in *L. monocytogenes*. Of these, *prfA*P1 and *prfA*P2 are intragenic promoters located upstream of the *prfA* gene on the LPI-1 that are activated by the vegetative sigma factor A (for *prfA*P1) and both sigma factor A and SigB (for *prfA*P2) [192]. However, the biological significance of SigB regulation of *prfA* expression has not been easy to establish [19]. The current hypothesis is that SigB regulation through the *prfA*P2 promoter may facilitate the downregulation of *prfA* transcription [19,28]. The repression is postulated to keep a basal level of PrfA that allows for a sensitive and rapid shift from the avirulent state to a virulent state once inside host cells [28]. Thus far, the main mechanism for the upregulation of *prfA* transcription inside host cells appears to be the positive autoregulatory feedback loop through the *prfA*P3 promoter [19]. The signal that triggers the upregulation of *prfA* transcription inside host cells remains unclear. One established observation is the role of temperature in the *prfA* expression [193,194]. In *L. monocytogenes*, a thermosensor located in the 5’-untranslated region (5′-UTR) of the *prfA* mRNA transcript allows for the translation of transcript at 37 °C, while preventing translation at temperatures <30 °C [195].

*The metabolic regulator CodY as a link between SigB and PrfA networks:* CodY is a Gram-positive bacterial metabolic regulator that responds to intracellular Guanosine-5’-triphosphate (GTP) concentration as an indicator of nutrient stress [196]. Under conditions of nutrient availability, CodY represses *sigB* expression, while activating the stress response regulator under conditions of nutrient stress [179]. Apart from GTP as the nutrient stress signal, CodY also responds to the cellular concentrations of branched chain amino acids (BCAA) [180]. Due to the low BCAA concentrations in host cells during infection, CodY plays a critical role in the biosynthesis of BCAAs through the upregulation of the *ilv* (isoleucine, leucine and valine (ILV)) operon [180]. Simultaneously, CodY results in an increased expression of virulence gene expression through a direct upregulation of *prfA*, while also causing an increased *sigB* expression [179]. Thus, the metabolic regulator acts as a link between *L. monocytogenes* metabolism, stress response, and pathogenesis (Figure 5) [178,197].

*Glutathione allosteric activation of PrfA as an indirect link between SigB and PrfA networks:* Similar to other Crp/Fnr family proteins, PrfA requires a co-factor to improve its DNA binding at promoter sequences [178]. In recent years, the tripeptide glutathione (GSH) has been identified as a post-translational activator of PrfA through allosteric mechanisms [198,199]. In addition to the direct role of SigB in the transcription of *prfA*, the stress response regulator also plays some indirect roles in stimulating *prfA* expression. Through the activity of the enzyme glutathione synthase (encoded by the *gshF* gene), *L. monocytogenes* can endogenously synthesize glutathione [200]. However, due to its antioxidant action, GSH is oxidized to GSSG. To maintain a healthy oxidative state, *L. monocytogenes* produces the SigB-regulated glutathione reductase (encoded by *lmo1433*) to modulate the GSH/GSSG ratio [17]. Thus, SigB contributes indirectly to PrfA activity by maintaining high levels of GSH reductase [12].

Along with the activation of *prfA*, there is also evidence to suggest that some stress response proteins of *L. monocytogenes* are involved in the post-transcriptional modification of some virulence factors [185,201]. For instance, Eshwar et al. [185] showed that *cspABD* deletion mutants could not produce the actin polymerization protein ActA, while Schärer et al. [201] made a similar observation with LLO production. Based on their observation, Eshwar et al. [185] hypothesized that the Csp proteins could be linked to the regulation of virulence gene expression at both transcriptional and post-transcriptional levels in *L. monocytogenes*.

## 6. Strain and Lineage Variability in Stress Response and Virulence

*L. monocytogenes* exhibits great heterogeneity within the species. The species is divided into four evolutionary lineages (designated lineages I, II, III, and IV), and 13 serotypes [202,203]. Additionally, using multilocus sequence typing (MLST) and whole-genome sequencing analysis, the species is divided into >100 clonal complexes (CCs) and sub-lineages (SLs) [204,205,206]. Among the lineages and serotypes, heterogeneity also exists with respect to virulence and ecological niche preferences [27,207]. Three serotypes belonging to lineage I (4b and 1/2b) and lineage II (1/2a) account for >95% of human listeriosis cases [208,209]. Furthermore, among the serotypes associated with human disease, serotype 4b strains are the most frequently implicated in severe clinical outcomes such as brain and placental infections [209,210]. On the other hand, lineage II strains are predominant in foods [209,211].

The molecular basis of *L. monocytogenes* strain and lineage virulence heterogeneity has been deciphered from the genetic differences between the so-called hypervirulent (serotype 4b, lineage I) and the hypovirulent (lineage II) strains [27,208]. Based on single nucleotide polymorphisms (SNPs) and multilocus genotyping (MLGT) using virulence (such as *inlA*, *inlB*; *hly*, *plcC*; *actA*) and *sigB* as well as whole-genome sequencing, several studies have shown that lineage II strains carry numerous mutations in their *inlA* genes that lead to premature stop codons and the production of truncated forms of inlA [212,213,214]. Consequently, despite their frequent occurrence in foods, lineage II strains are hypovirulent due to their inability to express a functional InlA protein needed for a successful systemic infection [27]. Apart from the core virulence genes encoded on LIPI-1 and the *inlAB* islet universally present in all *L. monocytogenes* strains, some lineage I strains harbor an extra set of virulence genes (the Listeria Pathogenicity Island 3 (LIPI-3)) [215,216]. LIPI-3 carries a gene cluster that includes the *llsA* gene conceding for the synthesis of Listeriolysin S (LLS), a bacteriocin whose function is to inhibit host microbiota during infection [216]. The absence of the LIPI-3 in the genomes of lineage II strains and its presence in the sub-lineages of lineage I (4b serotype) strains suggests that it is the main factor behind the hypervirulence of serotype 4b strains [27,204].

With respect to lineage II strains, their overrepresentation in the food environment is presumed to be a function of their ability to withstand environmental stress [203]. Available evidence shows that stress resistance phenotypes in *L. monocytogenes* are linked to genetic lineages, with lineage II strains particularly showing a better adaptation to environmental stresses such as salt, acid, and heat [217,218]. Using comparative genomics, of 174 clinical and food isolates, Pirone-Davies et al. [219] identified eight plasmid-borne genes uniquely associated with lineage II strains from food. These genes included cadmium resistance genes *cadA* and *cadC*, a multi-drug resistance gene *ebrB*, and a quaternary ammonium compound resistance gene *qac* [219]. Plasmid-harboring *L. monocytogenes* strains were found to be persistent in food processing environments and tolerant to benzalkonium chloride, elevated temperature, salinity, and acidic environments [220,221,222].

The near-total lack of association of lineage III strains with either foods or human infections is a subject that is still to be fully elucidated. A pan-genome analysis of 26 strains representing lineages I, II, and III, identified 86 disparately distributed genes highly conserved in lineages I and II genomes but highly divergent or absent in lineage III genomes [208]. Among the disparately distributed genes were genes involved in carbohydrate metabolism (phosphotransferase system (PTS)) and transcription factors [208]. Cerutti et al. [223] also identified genes for small regulatory RNAs that co-evolved with genes for pathogenicity and host interaction present in the genomes of lineage I and II strains but missing in the genomes of lineage III strains. Thus, reinforcing the hypothesis that lineage III strains evolved by loss of virulence and metabolic functions [208,223].

## 7. Conclusions and Future Perspectives

Adaptive stress tolerance responses play a critical role in the pathogenicity of *L. monocytogenes*. While stress adaptation is primarily a mechanism of environmental survival, the processes play a role in protecting the organism against the innate defence systems of the GIT. Furthermore, the transcriptional regulator of environmental stress adaptation, SigB, also has some concurrent effects on the expression of virulence factors that are also under the virulence regulator, PrfA. In parallel with the SigB regulatory effect on virulence genes, PrfA also exerts a synchronic effect on stress response genes for survival in the GIT. The regulatory overlap between the stress response and virulence serves as a point of coordination that facilitates a smooth transition from the avirulent saprophytic survival state to a virulent pathogenic state once inside the host.

Apart from the overlapping functions, direct and indirect interactions between the two transcriptional regulators account for the intricate link between the regulatory networks for stress response and virulence. While a significant amount of information is now available on the expressional crosstalk between SigB and PrfA as the respective central regulators of stress response and virulence, the full understanding of these molecular interactions is still elusive. As a prominent foodborne pathogen, an understanding of the molecular basis of *L. monocytogenes* stress survival and its influence on pathogenesis is critical to the identification of potential targets for the control of the pathogen through the interruption of its transmission and infection cycle.

## Figures and Tables

**Table 1:** Stress response proteins and virulence factors expressed by *L. monocytogenes* at different stages of the infection cycle.

Stage of Infection Cycle	Stress Response Proteins/Virulence Factor	Function	Transcriptional Regulator	References
Intestinal phase	GadD2T2	Glutamate decarboxylase for survival of stomach acidity	SigB	[104]
	OpuC	Carnitine transporter for osmotic stress survival	SigB	[116]
	Bsh	Bile salt hydrolase for bile stress survival	SigB and PrfA	[157,184]
	BilE	Bile exclusion system for bile stress survival	SigB and PrfA	[158]
	InlA	Adhesion and invasion of enterocytes	SigB and PrfA	[39,168]
	InlB	Adhesion and invasion of enterocytes	SigB and PrfA	[39]
	ActA	Bacterial aggregation and intestinal colonization	SigB and PrfA	[26,189]
Intracellular phase	LLO	Primary vacuole lysis	PrfA	[46]
	PI-PLC	Primary vacuole lysis	PrfA	[53]
	PC-PLC	Secondary vacuole lysis	PrfA	[54]
	ActA	Intracellular motility and cell-to-cell spread	PrfA	[56,190]

## Data Availability

Not applicable.
